# Research progress in endothelial cell injury and repair

**DOI:** 10.3389/fphar.2022.997272

**Published:** 2022-09-13

**Authors:** Yongpan Huang, Chong Song, Jianbin He, Min Li

**Affiliations:** ^1^ Medicine School, Changsha Social Work College, Changsha, Hunan, China; ^2^ Department of Respiratory and Critical Care Medicine, The First People’s Hospital of Huaihua, Affiliated to University of South China, Huaihua, Hunan, China

**Keywords:** endothelial cells, endothelial injury, endothelial repair, inflammatory response, oxidative stress, endothelial progenitor cells

## Abstract

Endothelial cells, which are important metabolic and endocrine cells, play an important role in regulating vascular function. The occurrence and development of various cardiovascular and cerebrovascular diseases are associated with endothelial dysfunction. However, the underlying mechanism of vascular endothelial injury is not fully understood. It has been reported that the mechanism of endothelial injury mainly involves inflammation and oxidative stress. Moreover, endothelial progenitor cells are regarded as important contributors in repairing damaged endothelium. Multiple interventions (including chemical drugs and traditional Chinese medicines) exert endothelial protection by decreasing the release of inducing factors, suppressing inflammation and oxidative stress, and preventing endothelial cell senescence.

## Introduction

Vascular endothelial cells are a single layer of flat epithelial cells located on the inner surface of the vascular lumen. They are distributed throughout large blood vessels and microvessels, and are important metabolic and endocrine organs. Vascular endothelial cells maintain circulatory stability, regulate vascular tone, and play an important role in anticoagulation and prevention of thrombosis (Bach, 2015). Vascular endothelial injury is seen in a variety of cardiovascular and cerebrovascular diseases, such as atherosclerosis, hypertension, and diabetic vascular disease, and is considered the initiating link of these diseases. Therefore, it is important to explore the factors and mechanisms of damaged endothelium, and to study the role and mechanism of changes in secreted active substances (Gimbrone and Garcia-Cardena, 2016). Endothelial cells possess the ability to proliferate and repair cell damage. Thus, endothelial cells are always in the dynamic process of being damaged and resisting damage, i.e., being repaired (Mazini et al., 2020). Considering the important role of vascular endothelium in the occurrence and development of dilemmas, the idea of interventions (drugs and biologics) to prevent and treat diseases is to facilitate the protection of endothelium and promote its repair (Miyamoto et al., 2014; Meng et al., 2018).

### Endothelial injury and cardiovascular and cerebrovascular diseases

Clinical studies and animal experiments have shown that a variety of cardiovascular and cerebrovascular diseases (chronic cardiac insufficiency, diabetic vascular complications, stroke) are accompanied by endothelial cell dysfunction (Jara and Mezzano, 2008; Moon, 2021). In clinical studies, surface high-frequency ultrasound may be used to detect brachial artery blood flow–mediated endothelium-dependent dilation (FMD) and non–endothelium-dependent diastolic function (nitroglycerin-mediated dilation, NMD) to detect nitric oxide in blood (nitric oxide, NO), von Willebrand factor (vWF), asymmetric dimetylarginine (ADMA), and other indirect indicators of endothelial function (Arslan et al., 2017; Mortensen et al., 2019; Stepanova et al., 2019). The morphological changes of endothelial injury have been observed and endothelial NO levels and endothelial NO synthase (eNOS) activity were detected in different experimental animal models (hypertension, atherosclerosis, diabetes) (Kietadisorn et al., 2012).

It has been revealed that various factors, such as oxidized low-density lipoprotein (ox-LDL), hyperglycemia, homocysteine (Hcy), hypoxia, hydrogen peroxide (H_2_O_2_), and reactive aldehydes, induce endothelial injury. This has been confirmed by exogenous application of these factors in animal experiments and cultured endothelial cells to directly damage the endothelium (endothelial cells) (Li et al., 2016; Caliceti et al., 2017; Nègre-Salvayre et al., 2017; Yang et al., 2020).

### Mechanism of endothelial cell injury

The mechanism of vascular endothelial cell injury is not fully understood. During the process of vascular endothelial injury, there are changes in vasodilation function, abnormal production and secretion of active substances, energy metabolism disorders, and morphological changes, and the underlying pathophysiological mechanism mainly involves inflammatory response and oxidative stress (Schinzari et al., 2017).

#### Inflammation and endothelial cell injury

A large number of studies have confirmed that the underlying pathophysiological mechanism of cardiovascular diseases, including atherosclerosis, involves inflammation (Zhong et al., 2019). Literature reports have demonstrated that atherosclerosis is a chronic inflammatory disease (Ross, 1999), with the characteristic of inflammatory cell infiltration and secretion of various inflammatory factors, including TNF-α, IL-6, and IL-1β (Fan et al., 2013; Jiang et al., 2019). Uncontrolled inflammation is also found in other metabolic diseases, including diabetes-induced vascular inflammation (Stepanova et al., 2019).

In addition to directly attacking vascular endothelium, oxidative stress contributes to inducing inflammation (Domingueti et al., 2016; Bai et al., 2020), which has been confirmed in a variety of cardio- and cerebrovascular diseases, such as atherosclerosis (e.g., ox-LDL), diabetes (e.g., high glucose and glycosylation end products), and hypertension (angiotensin II [Ang II]) (Pleskovič et al., 2017; Solis et al., 2021). Further research has shown that the mechanism of inflammatory response involves a variety of microRNAs (miR-126, miR-155, miR-221/222, miR-31, miR-17-3p, miR-10a, miR-663, miR-125a-5p, and miR-125b-5p) by regulating downstream target proteins (such as VCAM-1, RGS16, Ets-1, AT1R, E-selectin, ICAM-1, MAP3K7, and βTRC) (Forouzanfar and Asgharzade, 2020; Wu et al., 2020; Luan et al., 2022).

Atherosclerosis and other cardiovascular and cerebrovascular diseases show endothelial cell aging, and endothelial cells can secrete a series of inflammatory factors (such as TNF-α, IL-1β, IL-2, IL-6, IL- 8, RANTES, ICAM, VCAM), known as “aging inflammation,” which further aggravates endothelial injury (Montgolfier, 2019).

The following functional proteins have been investigated in the context of inflammatory response: ① NF-κB: It is the central link and common pathway of inflammatory response during endothelial injury. Many stimuli can activate the NF-κB signal transduction pathway and induce upregulation of the gene expression of inflammation-related cytokines. Recent studies have shown that NF-κB activity is regulated by epigenetics, such as PCB upregulation through epigenetics NF-κB subunit p65 expression induces endothelial inflammation (Zhu and Hou, 2021). ② High-mobility group protein 1 (HMGB1): It is a new type of inflammatory mediators and is associated with cardiovascular diseases (atherosclerosis, acute coronary syndrome, pulmonary hypertension) and closely related diseases (Jeong et al., 2019; Wahid et al., 2021). In the development of atherosclerosis, HMGB1 mediates the expression of proinflammatory mediators of endothelial cells during the initial stage of plaque formation, including TNF-α, IL-8, MCP-1, adhesion molecules (ICAM-1, VCAM-1), MIP-1α, and MIP-1β (Hebbel et al., 2020). *In vitro* experiments have shown that HMGB1 induces inflammatory responses through the TLR4 and IRF3 pathways (Florim et al., 2020). ③ Inflammasome: It is a newly discovered large molecular multiprotein complex with a molecular weight of 100 kDa, which is involved in atherosclerosis, ischemia–reperfusion injury, and type 2 diabetes. IL-1β is regarded as a pivotal inflammatory mediator, and its activation and secretion are regulated by inflammasomes (Palomo et al., 2015; Yu, 2016). In the process of inflammatory response, IL-1β induces the binding of intracellular Pro-IL-1β and inflammasome-related protein nucleotides. Increased synthesis of nucleotide-binding NLRP3 induces inflammasome assembly and activates Caspase-1, which cleaves Pro-IL-1β to generate activated IL-1β (Dinarello, 2011; Fahey and Doyle, 2019).

#### Oxidative stress and endothelial cell injury

It has been demonstrated that oxidative stress is an important mechanism involved in endothelial injury in atherosclerosis, diabetes, hypertension, and myocardial infarction (Ungvári et al., 2005; Husain et al., 2015). Various factors such as ox-LDL, Ang II, ADMA, hypoxia, high glucose, and reactive aldehydes can induce the generation of reactive oxygen species (ROS) (superoxide anion (O^2−^), H_2_O_2_, hydroxyl free (OH), hypochlorous acid (HOCl), and peroxynitrite (ONOOO-)), through direct or indirect injury to endothelial cells (Shaw et al., 2014). It is worth mentioning that the eNOS inhibitor ADMA competitively inhibits NOS and decouples it, so that it no longer catalyzes the production of NO by L-arginine, but induces O^2−^ production to promote oxidative stress (Jiang et al., 2006; Tan et al., 2007; Yuan et al., 2015). There is accumulating evidence that the causes of ROS accumulation are associated with the following elements: ① decreased activity of ROS-scavenging enzymes, such as superoxide dismutase, catalase, and glutathione peroxidase (O'Flaherty, 2019); ② increased activity of enzymes that catalyze the generation of ROS, such as peroxidase, xanthine oxidase, monoamine oxidase, and NADPH oxidase. Among them, peroxidase is a class of heme-containing enzymes that catalyzes H_2_O_2_ (weak oxidant) into HOCl (Awad et al., 2018). Previous studies on peroxidase have focused on myeloperoxidase (MPO), which is expresses in neutrophils and monocytes. In recent years, an isoenzyme of MPO has been discovered, which is 44.5% identical to MPO. In addition to being present in the heart, liver, and pancreas, it is highly expressed in vascular endothelial cells and vascular smooth muscle cells, which is why it is also known as vascular peroxide. Changes in the activity of vascular peroxidase (VPO) are closely related to endothelial injury in atherosclerosis, diabetes, and myocardial I/R injury (Bai et al., 2011; Awad et al., 2018).

Vascular aging often occurs in atherosclerosis, diabetes, coronary heart disease, and other cardiovascular and cerebrovascular diseases (Ma et al., 2013; Ishii et al., 2020). Hypoxia, ox-LDL, high glucose, and other factors can increase the expression of aging-related proteins such as p53, thereby resulting in endothelial cell aging (Tian and Li, 2014). Recently, it has been shown in diabetic rats and endothelial cells in a high glucose–induced injury model that the expression of VPO1 is upregulated, and that endothelial cells are senescent (Liu et al., 2015). Silencing the *VPO1* gene could significantly attenuate endothelial senescence. The exogenous application of HOCl could directly induce endothelial senescence, which suggests that the VPO1/HOCl pathway plays an important role in oxidative stress–induced endothelial cell aging (Yang et al., 2017). There are several lines of evidence that various factors, including oxidative stress, DNA damage, and genotoxic drugs, could induce cell senescence (Shokrzadeh et al., 2021). The mechanism involves regulating senescence-related miRNAs, which in turn regulate the expression of downstream target proteins and promote ROS generation, thereby leading to vascular aging. Overexpression of miR-146a in endothelial cells could significantly inhibit the expression of NADPH oxidase 4, and reduce the generation of ROS and endothelial senescence (Xiao et al., 2021). miR217 and miR-34a could cause downregulation on silent information regulator 1 (SIRT1) mRNA and protein, weaken antioxidant capacity, and deteriorate vascular endothelial aging (Zhang et al., 2020; Matacchione et al., 2021).

Previous studies have confirmed that ROS can promote endothelial cell morphological damage and induce apoptosis (Strilic et al., 2016). Necroptosis (also known as programmed necrosis) is a newly discovered type of cell death, which is found in the pathological process of I/R injury in the heart, kidney, brain, and retina. The main mechanism involves the interaction of TNF-α with TNF receptor 1 on the cell surface, which is mediated by the RIP1/RIP3/MLKL necrosis complex. Recent studies have shown that a variety of tumor cells (human lung adenocarcinoma cell line A549, human neuroblastoma cell SH-SY5Y) co-cultured with endothelial cells can induce endothelial cell necroptosis (Lubrano and Balzan, 2014; Chen et al., 2016).

### Repair and mechanism of damaged vascular endothelium

Endothelial cells possess the ability to self-proliferate and repair. Vascular endothelial injury not only affects the function of the vascular barrier and the regulatory function and secretory function of the vasodilator response, but also weakens the repair ability. Endothelial cells can slow down or even stop their own natural aging process by reducing or preventing the damage of endothelial cells and facilitating the repair of the damaged endothelial cells.

#### Endothelial progenitor cells and endothelial repair

Endothelial progenitor cells (EPCs) are stem cells that are homed to angiogenesis tissues. They can differentiate and proliferate into mature endothelial cells, and exert an important role in endothelial repair and angiogenesis. Several studies have demonstrated that the pathogenesis of various cardiovascular diseases (such as atherosclerosis and pulmonary hypertension) is associated with EPCs aging (Zhou et al., 2010a; Zhou et al., 2010b). After EPCs aging, their migration, adhesion function, and blood vessel formation ability are all reduced, resulting in weakened endothelial repair ability.

There are now three basic ways to treat endothelial damage with EPC: ①Transplantation of EPCs to endothelial injury sites to promote endothelial tissue regeneration and repair. It has been confirmed that the injection of EPCs into mice could significantly improve the damage of hepatic sinusoidal endothelial cells and hepatocytes while reducing the secretion of IL-6 and TNF-α, inhibiting platelet activation, and improving liver function (Qiao et al., 2015). ② The introduction of certain genes, such as calcitonin gene–related peptide (CGRP), into EPCs to enhance the protective effect of EPCs on endothelial cells. In animal experiments, EPCs transfected with CGRP in rats with pulmonary arterial hypertension can significantly improve pulmonary hypertension and reverse pulmonary vascular remodeling (Zhao et al., 2007). As shown *in vitro*, transfection of damaged EPCs into β2 adrenergic receptors could significantly improve the repair ability of EPCs on vascular endothelium (Ke et al., 2016). ③ Some drugs such as low-dose aspirin, resveratrol, rosiglitazone, pyrrolizone, and evodiamine can delay EPCs aging. For example, resveratrol-based derivative BTM-0512 inhibits EPCs aging in diabetic rats, and its mechanism of action involves the SIRTl-DDAH2/ADMA pathway (Yuan et al., 2015). CGRP mediates evodiamine and inhibits AngⅡ-induced EPC aging, and its mechanism is related to the upregulation of Klotho gene (Ming et al., 2016). Cisceral lipin delays ox-LDL–induced EPCs aging and upregulation of SIRT1 is involved, and the underlying mechanism involves the PI3K/Akt/ERK pathway (Xu et al., 2021).

#### Drugs and endothelial protection

**FIGURE 1:**
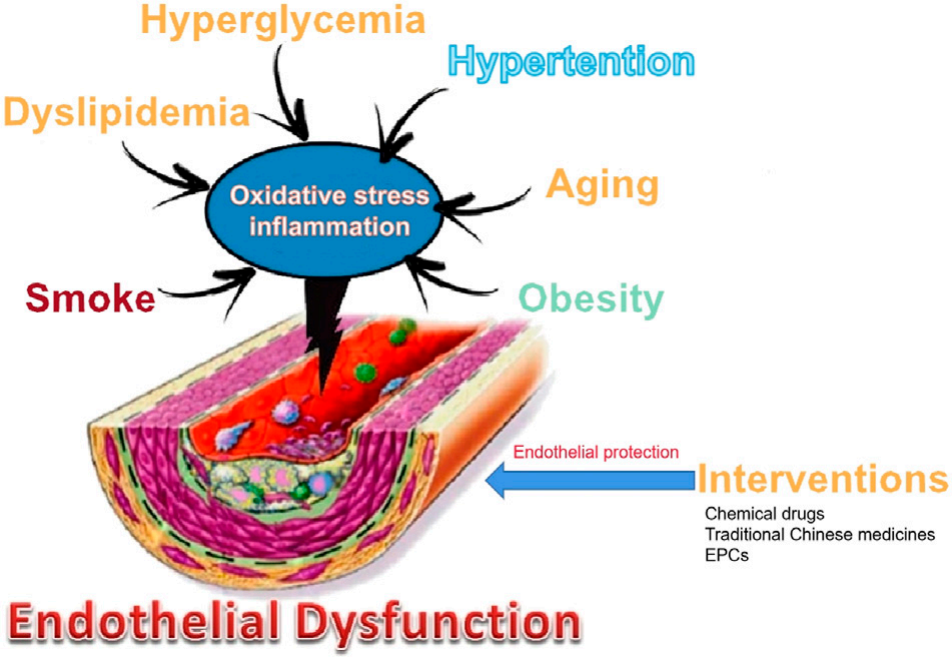
Main vascular risk factors and their involvement in endothelial dysfunction and intervention.

Clinical studies and animal experiments have shown that many drugs, including chemical drugs and traditional Chinese medicines, have protective effects on endothelial cells, but the underlying mechanisms are not yet fully understood (Yang et al., 2005). This is because different studies have discussed the various entrance points of the mechanism of pharmacological protection of vascular endothelium, apart from the complexity of the pathological mechanism of vascular endothelial injury (Figure 1). There are various mechanisms by which drugs may protect the vascular endothelium: ① Reducing the generation of factors that induce endothelial cell damage: For example, lipid-lowering drugs, hypoglycemic drugs, and anti-myocardial ischemia drugs can reduce the production of blood sugar, ox-LDL, ROS, and inflammatory factors. Folic acid can inhibit Hcy production, which is an adjunct therapy for hyperhomocysteinemia-type hypertension, helps protect vascular endothelium, and reduces the incidence of stroke (Haklar et al., 1998). L-arginine can competitively prevent ADMA from inhibiting eNOS. Vascular tension invertase inhibitors can reduce the production of Ang II. ② Reducing the formation of ROS by oxidative stress inhibitors such probucol, vitamin E, and tanshinone IIA (Tang et al., 2011; Jia et al., 2012; Tsai et al., 2014). ③Inhibiting the inflammatory response: For example, aspirin, fibrate lipid-lowering drugs, and resveratrol methyl derivatives may inhibit the production of inflammatory factors (Price et al., 2012; Wang et al., 2014; Jeong et al., 2015; Pan et al., 2016; Han et al., 2020). ④ Delaying aging of endothelial cells and EPCs: For example, rosiglitazone, evodiamine, and simvastatin can inhibit endothelial aging (Fraineau et al., 2015). EPC-based transcription is regulated by epigenetic regulation, including noncoding RNA (microRNA and IncRNA), DNA methylation, histone modification (histone methylation, acetylation, and deacetylation), and some compounds (such as peptide compound inhibitor 5-azacytidine). Epigenetics increases the proliferation and migration of EPCs and enhances the ability to repair blood vessels (Fraineau et al., 2015).

## Conclusion

Vascular endothelial injury is the initiating link of various cardiovascular and cerebrovascular diseases. In addition to the changes in its own morphology and function, endothelial injury causes endothelial cells to secrete endogenous active substances and affect vascular smooth muscle, which affects vasodilation. It has also been demonstrated that a number of variables, including hypoxia and others, can cause endothelial interstitial change and promote vascular remodeling (Tian and Li, 2014). The exact mechanism of endothelial cell injury is not fully understood. Inflammation and oxidative stress are known as important pathophysiological mechanisms of endothelial injury. It is known that there is an interaction between inflammation and oxidative stress, but the network relationship of their interaction and its key molecules are yet to be elucidated. The aging and regulation mechanisms of endothelial cells and EPCs also need to be further explored. With the deepening of research on endothelial injury and repair, new targets for protecting vascular endothelium may be discovered, which will provide new ideas to find drugs to protect damaged endothelium. It has been proven that a variety of traditional Chinese medicines have protective effects on vascular endothelium, and the separation and purification of active ingredients and monomers in traditional Chinese medicine will be an important way to develop drugs for vascular endothelial protection.
