# Supplementary material for: Research progress in endothelial cell injury and repair
Source: Front Pharmacol. 2022 Sep 13;13:997272. doi: 10.3389/fphar.2022.997272 (PMC9513221; doi:10.3389/fphar.2022.997272)
Supplement: Supplementary file 1 [file Table1.DOCX]

**Research progress in endothelial cell injury and repair**

Yongpan Huang^1^, Chong Song^1^, Jianbin He^2^, Min Li^1^

^1^Medicine School, Changsha Social Work College, Changsha, Hunan 410004;

^2^Department of Respiratory and Critical Care Medicine, The First People's Hospital of Huaihua, affiliated to University of South China, Huaihua, Hunan, 418000;

*Correspondence to:*

Pro. Jianbin He, Department of Respiratory and Critical Care Medicine, The First People's Hospital of Huaihua, affiliated to University of South China, Huaihua, Hunan, 418000.

E-mail: [hjb0919hh@163.com](mailto:hjb0919hh@163.com)

Pro. Min Li. Medicine School, Changsha Social Work College, Changsha, Hunan 410004

E-mail: 2330692557@qq.com

**Abstract**

Endothelial cells, which are important metabolic and endocrine cells, play an important role in regulating vascular function. The occurrence and development of various cardiovascular and cerebrovascular diseases are associated with endothelial dysfunction. However, the underlying mechanism of vascular endothelial injury is not fully understood. It has been reported that the mechanism of endothelial injury mainly involves inflammation and oxidative stress. Moreover, endothelial progenitor cells are regarded as important contributors in repairing damaged endothelium. Multiple interventions (including chemical drugs and traditional Chinese medicines) exert endothelial protection by decreasing the release of inducing factors, suppressing inflammation and oxidative stress, and preventing endothelial cell senescence.

**[Keywords]** endothelial cells; oxidative stress; endothelial repair; inflammation; endothelial progenitor cells

**Introduction**

Vascular endothelial cells are a single layer of flat epithelial cells located on the inner surface of the vascular lumen. They are distributed throughout large blood vessels and microvessels, and are important metabolic and endocrine organs. Vascular endothelial cells maintain circulatory stability, regulate vascular tone, and play an important role in anticoagulation and prevention of thrombosis (1). Vascular endothelial injury is seen in a variety of cardiovascular and cerebrovascular diseases, such as atherosclerosis, hypertension, and diabetic vascular disease, and is considered the initiating link of these diseases. Therefore, it is important to explore the factors and mechanisms of damaged endothelium, and to study the role and mechanism of changes in secreted active substances (2). Endothelial cells possess the ability to proliferate and repair cell damage. Thus, endothelial cells are always in the dynamic process of being damaged and resisting damage, i.e., being repaired (3). Considering the important role of vascular endothelium in the occurrence and development of dilemmas, the idea of interventions (drugs and biologics) to prevent and treat diseases is to facilitate the protection of endothelium and promote its repair (4,5).

**1 Endothelial injury and cardiovascular and cerebrovascular diseases**

Clinical studies and animal experiments have shown that a variety of cardiovascular and cerebrovascular diseases (chronic cardiac insufficiency, diabetic vascular complications, stroke) are accompanied by endothelial cell dysfunction (6,7). In clinical studies, surface high-frequency ultrasound may be used to detect brachial artery blood flow–mediated endothelium-dependent dilation (FMD) and non–endothelium-dependent diastolic function (nitroglycerin-mediated dilation, NMD) to detect nitric oxide in blood (nitric oxide, NO), von Willebrand factor (vWF), asymmetric dimetylarginine (ADMA), and other indirect indicators of endothelial function (8-10). The morphological changes of endothelial injury have been observed and endothelial NO levels and endothelial NO synthase (eNOS) activity were detected in different experimental animal models (hypertension, atherosclerosis, diabetes) (11).

It has been revealed that various factors, such as oxidized low-density lipoprotein (ox-LDL), hyperglycemia, homocysteine (Hcy), hypoxia, hydrogen peroxide (H_2_O_2_), and reactive aldehydes, induce endothelial injury. This has been confirmed by exogenous application of these factors in animal experiments and cultured endothelial cells to directly damage the endothelium (endothelial cells) (12-15).

2 **Mechanism of endothelial cell injury**

The mechanism of vascular endothelial cell injury is not fully understood. During the process of vascular endothelial injury, there are changes in vasodilation function, abnormal production and secretion of active substances, energy metabolism disorders, and morphological changes, and the underlying pathophysiological mechanism mainly involves inflammatory response and oxidative stress (16).

**2.1 Inflammation and endothelial cell injury**

A large number of studies have confirmed that the underlying pathophysiological mechanism of cardiovascular diseases, including atherosclerosis, involves inflammation (17). Literature reports have demonstrated that atherosclerosis is a chronic inflammatory disease [18], with the characteristic of inflammatory cell infiltration and secretion of various inflammatory factors, including TNF-α, IL-6, and IL-1β (19,20). Uncontrolled inflammation is also found in other metabolic diseases, including diabetes-induced vascular inflammation (8).

In addition to directly attacking vascular endothelium, oxidative stress contributes to inducing inflammation (21,22), which has been confirmed in a variety of cardio- and cerebrovascular diseases, such as atherosclerosis (e.g., ox-LDL), diabetes (e.g., high glucose and glycosylation end products), and hypertension (angiotensin II [Ang II]) (23,24). Further research has shown that the mechanism of inflammatory response involves a variety of microRNAs (miR-126, miR-155, miR-221/222, miR-31, miR-17-3p, miR-10a, miR-663, miR-125a-5p, and miR-125b-5p) by regulating downstream target proteins (such as VCAM-1, RGS16, Ets-1, AT1R, E-selectin, ICAM-1, MAP3K7, and βTRC) (25-27).

Atherosclerosis and other cardiovascular and cerebrovascular diseases show endothelial cell aging, and endothelial cells can secrete a series of inflammatory factors (such as TNF-α, IL-1β, IL-2, IL-6, IL- 8, RANTES, ICAM, VCAM), known as "aging inflammation," which further aggravates endothelial injury (28).

The following functional proteins have been investigated in the context of inflammatory response: ① NF-κB: It is the central link and common pathway of inflammatory response during endothelial injury. Many stimuli can activate the NF-κB signal transduction pathway and induce upregulation of the gene expression of inflammation-related cytokines. Recent studies have shown that NF-κB activity is regulated by epigenetics, such as PCB upregulation through epigenetics NF-κB subunit p65 expression induces endothelial inflammation (29). ② High-mobility group protein 1 (HMGB1): It is a new type of inflammatory mediators and is associated with cardiovascular diseases (atherosclerosis, acute coronary syndrome, pulmonary hypertension) and closely related diseases (30, 31). In the development of atherosclerosis, HMGB1 mediates the expression of proinflammatory mediators of endothelial cells during the initial stage of plaque formation, including TNF-α, IL-8, MCP-1, adhesion molecules (ICAM-1, VCAM-1), MIP-1α, and MIP-1β (32). In vitro experiments have shown that HMGB1 induces inflammatory responses through the TLR4 and IRF3 pathways (33). ③ Inflammasome: It is a newly discovered large molecular multiprotein complex with a molecular weight of 100 kDa, which is involved in atherosclerosis, ischemia–reperfusion injury, and type 2 diabetes. IL-1β is regarded as a pivotal inflammatory mediator, and its activation and secretion are regulated by inflammasomes (34,35). In the process of inflammatory response, IL-1β induces the binding of intracellular Pro-IL-1β and inflammasome-related protein nucleotides. Increased synthesis of nucleotide-binding NLRP3 induces inflammasome assembly and activates Caspase-1, which cleaves Pro-IL-1β to generate activated IL-1β (36, 37).

**2.2 Oxidative stress and endothelial cell injury**

It has been demonstrated that oxidative stress is an important mechanism involved in endothelial injury in atherosclerosis, diabetes, hypertension, and myocardial infarction (38,39). Various factors such as ox-LDL, Ang II, ADMA, hypoxia, high glucose, and reactive aldehydes can induce the generation of reactive oxygen species (ROS) (superoxide anion (O^2-^), H_2_O_2_, hydroxyl free (OH), hypochlorous acid (HOCl), and peroxynitrite (ONOOO-)), through direct or indirect injury to endothelial cells (40). It is worth mentioning that the eNOS inhibitor ADMA competitively inhibits NOS and decouples it, so that it no longer catalyzes the production of NO by L-arginine, but induces O^2-^ production to promote oxidative stress (41-43). There is accumulating evidence that the causes of ROS accumulation are associated with the following elements: ① decreased activity of ROS-scavenging enzymes, such as superoxide dismutase, catalase, and glutathione peroxidase (44); ② increased activity of enzymes that catalyze the generation of ROS, such as peroxidase, xanthine oxidase, monoamine oxidase, and NADPH oxidase. Among them, peroxidase is a class of heme-containing enzymes that catalyzes H_2_O_2_ (weak oxidant) into HOCl (45). Previous studies on peroxidase have focused on myeloperoxidase (MPO), which is expresses in neutrophils and monocytes. In recent years, an isoenzyme of MPO has been discovered, which is 44.5% identical to MPO. In addition to being present in the heart, liver, and pancreas, it is highly expressed in vascular endothelial cells and vascular smooth muscle cells, which is why it is also known as vascular peroxide. Changes in the activity of vascular peroxidase (VPO) are closely related to endothelial injury in atherosclerosis, diabetes, and myocardial I/R injury (45, 46).

Vascular aging often occurs in atherosclerosis, diabetes, coronary heart disease, and other cardiovascular and cerebrovascular diseases (47,48). Hypoxia, ox-LDL, high glucose, and other factors can increase the expression of aging-related proteins such as p53, thereby resulting in endothelial cell aging (49). Recently, it has been shown in diabetic rats and endothelial cells in a high glucose–induced injury model that the expression of VPO1 is upregulated, and that endothelial cells are senescent (50). Silencing the *VPO1* gene could significantly attenuate endothelial senescence. The exogenous application of HOCl could directly induce endothelial senescence, which suggests that the VPO1/HOCl pathway plays an important role in oxidative stress–induced endothelial cell aging (51). There are several lines of evidence that various factors, including oxidative stress, DNA damage, and genotoxic drugs, could induce cell senescence (52). The mechanism involves regulating senescence-related miRNAs, which in turn regulate the expression of downstream target proteins and promote ROS generation, thereby leading to vascular aging. Overexpression of miR-146a in endothelial cells could significantly inhibit the expression of NADPH oxidase 4, and reduce the generation of ROS and endothelial senescence (53). miR217 and miR-34a could cause downregulation on silent information regulator 1 (SIRT1) mRNA and protein, weaken antioxidant capacity, and deteriorate vascular endothelial aging (54,55).

Previous studies have confirmed that ROS can promote endothelial cell morphological damage and induce apoptosis (56). Necroptosis (also known as programmed necrosis) is a newly discovered type of cell death, which is found in the pathological process of I/R injury in the heart, kidney, brain, and retina. The main mechanism involves the interaction of TNF-α with TNF receptor 1 on the cell surface, which is mediated by the RIP1/RIP3/MLKL necrosis complex. Recent studies have shown that a variety of tumor cells (human lung adenocarcinoma cell line A549, human neuroblastoma cell SH-SY5Y) co-cultured with endothelial cells can induce endothelial cell necroptosis (57, 58).

**3 Repair and mechanism of damaged vascular endothelium**

Endothelial cells possess the ability to self-proliferate and repair. Vascular endothelial injury not only affects the function of the vascular barrier and the regulatory function and secretory function of the vasodilator response, but also weakens the repair ability. Endothelial cells can slow down or even stop their own natural aging process by reducing or preventing the damage of endothelial cells and facilitating the repair of the damaged endothelial cells.

**3.1 Endothelial progenitor cells and endothelial repair**

Endothelial progenitor cells (EPCs) are stem cells that are homed to angiogenesis tissues. They can differentiate and proliferate into mature endothelial cells, and exert an important role in endothelial repair and angiogenesis. Several studies have demonstrated that the pathogenesis of various cardiovascular diseases (such as atherosclerosis and pulmonary hypertension) is associated with EPCs aging (59,60). After EPCs aging, their migration, adhesion function, and blood vessel formation ability are all reduced, resulting in weakened endothelial repair ability.

There are now three basic ways to treat endothelial damage with EPC: ①Transplantation of EPCs to endothelial injury sites to promote endothelial tissue regeneration and repair. It has been confirmed that the injection of EPCs into mice could significantly improve the damage of hepatic sinusoidal endothelial cells and hepatocytes while reducing the secretion of IL-6 and TNF-α, inhibiting platelet activation, and improving liver function (61). ② The introduction of certain genes, such as calcitonin gene–related peptide (CGRP), into EPCs to enhance the protective effect of EPCs on endothelial cells. In animal experiments, EPCs transfected with CGRP in rats with pulmonary arterial hypertension can significantly improve pulmonary hypertension and reverse pulmonary vascular remodeling (62). As shown in vitro, transfection of damaged EPCs into β2 adrenergic receptors could significantly improve the repair ability of EPCs on vascular endothelium (63). ③ Some drugs such as low-dose aspirin, resveratrol, rosiglitazone, pyrrolizone, and evodiamine can delay EPCs aging. For example, resveratrol-based derivative BTM-0512 inhibits EPCs aging in diabetic rats, and its mechanism of action involves the SIRTl-DDAH2/ADMA pathway (64). CGRP mediates evodiamine and inhibits AngⅡ-induced EPC aging, and its mechanism is related to the upregulation of Klotho gene (65). Cisceral lipin delays ox-LDL–induced EPCs aging and upregulation of SIRT1 is involved, and the underlying mechanism involves the PI3K/Akt/ERK pathway (66).

**3.2 Drugs and endothelial protection**

Clinical studies and animal experiments have shown that many drugs, including chemical drugs and traditional Chinese medicines, have protective effects on endothelial cells, but the underlying mechanisms are not yet fully understood (67). This is because different studies have discussed the various entrance points of the mechanism of pharmacological protection of vascular endothelium, apart from the complexity of the pathological mechanism of vascular endothelial injury. There are various mechanisms by which drugs may protect the vascular endothelium: ① Reducing the generation of factors that induce endothelial cell damage: For example, lipid-lowering drugs, hypoglycemic drugs, and anti-myocardial ischemia drugs can reduce the production of blood sugar, ox-LDL, ROS, and inflammatory factors. Folic acid can inhibit Hcy production, which is an adjunct therapy for hyperhomocysteinemia-type hypertension, helps protect vascular endothelium, and reduces the incidence of stroke (68). L-arginine can competitively prevent ADMA from inhibiting eNOS. Vascular tension invertase inhibitors can reduce the production of Ang II. ② Reducing the formation of ROS by oxidative stress inhibitors such probucol, vitamin E, and tanshinone IIA (69-71). ③Inhibiting the inflammatory response: For example, aspirin, fibrate lipid-lowering drugs, and resveratrol methyl derivatives may inhibit the production of inflammatory factors (72-76). ④ Delaying aging of endothelial cells and EPCs: For example, rosiglitazone, evodiamine, and simvastatin can inhibit endothelial aging (77). EPC-based transcription is regulated by epigenetic regulation, including noncoding RNA (microRNA and IncRNA), DNA methylation, histone modification (histone methylation, acetylation, and deacetylation), and some compounds (such as peptide compound inhibitor 5-azacytidine). Epigenetics increases the proliferation and migration of EPCs and enhances the ability to repair blood vessels (78).

**Conclusion**

Vascular endothelial injury is the initiating link of various cardiovascular and cerebrovascular diseases. In addition to the changes in its own morphology and function, endothelial injury causes endothelial cells to secrete endogenous active substances and affect vascular smooth muscle, which affects vasodilation. It has also been demonstrated that a number of variables, including hypoxia and others, can cause endothelial interstitial change and promote vascular remodeling (49). The exact mechanism of endothelial cell injury is not fully understood. Inflammation and oxidative stress are known as important pathophysiological mechanisms of endothelial injury. It is known that there is an interaction between inflammation and oxidative stress, but the network relationship of their interaction and its key molecules are yet to be elucidated. The aging and regulation mechanisms of endothelial cells and EPCs also need to be further explored. With the deepening of research on endothelial injury and repair, new targets for protecting vascular endothelium may be discovered, which will provide new ideas to find drugs to protect damaged endothelium. It has been proven that a variety of traditional Chinese medicines have protective effects on vascular endothelium, and the separation and purification of active ingredients and monomers in traditional Chinese medicine will be an important way to develop drugs for vascular endothelial protection.

**Acknowledgments**

We thank LetPub (www.letpub.com) for its linguistic assistance during the preparation of this manuscript.

**Authors’ Contributions**

YPH analyzed, interpreted, and presented results for group discussions. YPH and CS provided rationale, background, framework, and feedback. All authors have approved the manuscript. All authors have agreed with both to be personally accountable for the author’s contributions and to ensure that questions related to the accuracy or integrity of any part of the work.

**Funding**

This work was supported by Hunan Natural Science Foundation (grant no. 2020JJ7090.

**Conflict of Interest**

There were no conflicts of interest to be declared.

**Disclaimer**

None.

**Data Availability**

The data used to support the findings are available from the corresponding author upon request.

**References**

**Research progress in endothelial cell injury and repair**

Yongpan Huang^1^, Chong Song^1^, Jianbin He^2^, Min Li^1^

^1^Medicine School, Changsha Social Work College, Changsha, Hunan 410004;

^2^Department of Respiratory and Critical Care Medicine, The First People's Hospital of Huaihua, affiliated to University of South China, Huaihua, Hunan, 418000;

*Correspondence to:*

Pro. Jianbin He, Department of Respiratory and Critical Care Medicine, The First People's Hospital of Huaihua, affiliated to University of South China, Huaihua, Hunan, 418000.

E-mail: [hjb0919hh@163.com](mailto:hjb0919hh@163.com)

Pro. Min Li. Medicine School, Changsha Social Work College, Changsha, Hunan 410004

E-mail: 2330692557@qq.com

**Abstract**

Endothelial cells are important metabolic and endocrine organs and play an important role in regulating vascular function. The occurrence and development of various cardiovascular and cerebrovascular diseases are associated with endothelial dysfunctions. However, underlying mechanism of vascular endothelial injury is not fully understood. It has been reported that the mechanism of endothelial injury mainly involves inflammation and oxidative stress. Moreover, endothelial progenitor cells are regarded as important contributors in repairing damaged endothelium. Multiples of interventions (including chemical drugs and traditional Chinese medicines) exert endothelial protection by decreasing the releases inducing factors, suppressing inflammation and oxidative stress, and preventing endothelial cell senescence.

**[Key words]** endothelial cells; oxidative stress; endothelial repair; inflammation; endothelial progenitor cells

**Introduction**

Vascular endothelial cells are a single layer of flat epithelial cells, which are located on the inner surface of the vascular lumen, and are distributed throughout the body's large blood vessels to various organs and microvessels，which are one of the body's important metabolic and endocrine organs. Vascular endothelial cells maintain circulatory stability, regulate vascular tone, Anticoagulation and prevention of thrombosis play an important role (1). Vascular endothelial injury is seen in a variety of cardiovascular and cerebrovascular diseases such as atherosclerosis, hypertension, and diabetic vascular disease. It is considered to be the initiating link of these diseases. Therefore, efforts are made to explore the factors and mechanisms of damaged endothelium, as well as to study the role and mechanism of changes in active substances secreted (2); on the other hand, endothelial cells possess the ability to proliferate and are also organs that could repair the cell damage, so endothelial cells are always in place during the dynamic process of being damaged and resisting damage (repair) (3), considering important role of vascular endothelium in the occurrence and development of dilemmas, the ideas of seeking for interventions (drugs and biologics) to prevent and treat diseases is to facilitate the protection on endothelium and repair (4,5).

**1 Endothelial injury and cardiovascular and cerebrovascular diseases**

Clinical studies and animal experiments have shown that a variety of cardiovascular and cerebrovascular diseases (chronic cardiac insufficiency, diabetic vascular complications, stroke) are accompanied by endothelial cell dysfunction (6,7). In clinical studies, the use of surface high-frequency ultrasound to detect brachial artery blood flow-mediated endothelium-dependent dilation (FMD) and non-endothelial dependence diastolic function (nitroglycerin-mediated dilation, NMD) to detect nitric oxide in blood (Nitric oxide, NO), von wilebrand factor (vWFF), asymmetric dimetylarginine (ADMA) and other indirect evaluation of endothelial function (8-10). In animal experiments, the morphological changes of endothelial injury were observed and endothelial NO levels and endothelial nitric oxide synthase (eNOS) activity were detected in different experimental animal models (hypertension, atherosclerosis, diabetes) (11).

It has been revealed that various factors such as oxidized low density lipoprotein (ox-LDL), hyperglycemia, homocysteine (Hcy), hypoxia, hydrogen peroxide (H_2_O_2_), reactive aldehydes are all important factors to induce endothelial injury, which has been confirmed by exogenous application of these factors *in vivo* animals and cultured endothelial cells to directly damage the endothelium (endothelial cells) (12-15).

2 **Mechanism of endothelial cell injury**

The mechanism of vascular endothelial cell injury is not fully understood. During the process of vascular endothelial injury, changes in vasodilation function, abnormal production and secretion of active substances, energy metabolism disorders and morphological changes, the underlying pathophysiological mechanism mainly involves inflammatory response and oxidative stress (16).

**2.1 Inflammation and endothelial cell injury**

A large number of investigations have confirmed that the underlying pathophysiological mechanisms of cardiovascular diseases, including atherosclerosis, which involved inflammation (17). Literature reports have demonstrated atherosclerosis as a chronic inflammatory disease [18], with the characteristic of inflammatory cell infiltration and secretion of various inflammatory factors, including TNF-α, IL-6, IL-1β (19,20). Uncontrolled inflammation is also seen in other metabolic diseases, including diabetes-induced vascular inflammation (8).

In addition to directly attacking the vascular endothelium, oxidative stress is also a contributor in inducing inflammation (21,22), which confirmed in a variety of cardio- and cerebrovascular diseases, such as atherosclerosis (ox-LDL, etc.), diabetes (high glucose, glycosylation, etc.) End products, etc.), hypertension [angiotensin II (Ang II) induce oxidative stress and then induce inflammatory response (23,24). Further research found that the mechanism of inflammatory response involves a variety of microRNAs (miR-126, miR-155, miR-221/222, miR-31, miR-17-3p, miR-10a, miR -663, miR-125a-5p and miR-125b-5p), by regulating downstream target proteins (such as VCAM-1, RGS16, Ets-1, AT1R, E-selectin, ICAM-1, MAP3K7 and βTRC) (25-27).

Atherosclerosis and other cardiovascular and cerebrovascular diseases show endothelial cell aging, and endothelial cells can secrete a series of inflammatory factors (such as TNF-α, IL-1β, IL-2, IL-6, IL- 8, RANTES, ICAM, VCAM), known as "aging inflammation", further aggravates endothelial injury (28).

The following functional proteins have continuously been better understood as the mechanism of the inflammatory response has been investigated:①NF-κB: it is the central link and common pathway of inflammatory response during endothelial injury. Many stimuli can activate the NF-κB signal transduction pathway and induce the up-regulation of the gene expression of inflammation-related cytokines. Recent studies have found that NF-κB activity is regulated by epigenetics, such as PCB upregulation through epigenetics NF-κB subunit p65 expression induces endothelial inflammation (29). ②High mobility group protein 1 (HMGB1): It is a new type of inflammatory mediator and is associated with cardiovascular diseases (atherosclerosis, acute coronary syndrome, pulmonary hypertension) and closely related (30, 31); in the development of atherosclerosis, HMGB1 mediates the expression of proinflammatory mediators of endothelial cells during the initial stage of plaque formation, including TNF-α, IL-8, MCP-1, adhesion molecules (ICAM-1, VCAM-1) and MIP-1α, MIP-1β (32). *In vitro* experiments showed that HMGB1 induces inflammatory responses through the TLR4 and IRF3 pathways (33). ③Inflammasome: It is a newly discovered large molecular multiprotein complex with a molecular weight of 100 kDa, which are involved in atherosclerosis, ischemia-reperfusion injury, type 2 diabetes. IL-1β is regarded as a pivotal inflammatory mediator, and its activation and secretion are regulated by inflammasomes. In the process of inflammatory response, IL-1β induces the binding of intracellular Pro-IL-1β and inflammasome-related protein nucleotides Increased synthesis of nucleotide-binding NLRP3 induces inflammasome assembly and activates Caspase-1, which cleaves Pro-IL- 1β generates activated IL-1β (35, 36).

**2.2 Oxidative stress and endothelial cell injury**

Studies have demonstrated that oxidative stress is an important mechanism for endothelial injury in atherosclerosis, diabetes, hypertension, myocardial infarction. It has been revealed that various factors such as ox-LDL, Ang II, ADMA, hypoxia, high sugar, and reactive aldehyde can induce the generation of ROS (superoxide anion (O^2-^), H_2_O_2_, hydroxyl free (OH), hypochlorous acid (HOCl) and peroxynitrite (ONOOO-)), through direct or indirect injury to endothelial cells (37). It is worth mentioning that the eNOS inhibitor ADMA competitively inhibits NOS and decouples it, that is, it no longer catalyzes the production of NO by L-arginine, but induces O^2-^ production to promote oxidative stress (38-40). Accumulating evidences have shown that the causes of ROS accumulation are associated with following elements: ①Decreased activity of ROS scavenging enzymes such as superoxide dismutase, catalase, and glutathione peroxidase (41); ②Enzymes that catalyze the generation of ROS, such as peroxidase, xanthine oxidase, monoamine oxidase and NADPH oxidase, have increased activity. Among them, peroxidase is a class of enzymes containing heme, which can catalyze H_2_O_2_ (weak oxidant) into HOCl (42). Previous studies on peroxidase have focused on myeloperoxidase (MPO), which is expresses in neutrophils and monocytes. In recent years, an isoenzyme of MPO has been discovered, which is 44.5% identical to MPO. In addition to being present in the heart, liver and pancreas, it is also highly expressed in vascular endothelial cells and vascular smooth muscle cells, called vascular peroxide. Vascular peroxidase (VPO), changes in the activity of this enzyme are closely related to endothelial injury in atherosclerosis, diabetes, myocardial I/R injury (42, 43).

Vascular aging often occurs in atherosclerosis, diabetes, coronary heart disease and other cardiovascular and cerebrovascular diseases. Hypoxia, ox-LDL, high glucose and other factors can increase the expression of aging-related proteins such as p53, resulting in endothelial cell aging (45). Recently, in diabetic rats and endothelial cells with high glucose-induced injury model, the expression of VPO1 is up-regulated, and the endothelial cells were senescent. Silencing the VPO1 gene could significantly attenuate endothelial senescence. The exogenous application of HOCl could directly induce endothelial senescence, which suggests that the VPO1/HOCl pathway plays an important role in oxidative stress-induced endothelial cell aging (46). Several lines of studies demonstrated that various factors including oxidative stress, DNA damage, and genotoxic drugs could induce cell senescence. The mechanism involves regulating senescence-related micRNAs, which in turn regulate the expression of downstream target proteins and promote ROS generation, and leads to vascular aging. Overexpression of miR-146a in endothelial cells could significantly inhibit the expression of NADPH oxidase 4, reduce the generation of ROS and endothelial senescence. miR217 and miR-34a could cause down-regulation on silent information regulator 1 (SIRT1) mRNA and protein, weaken the anti-oxidative stress and deteriorate vascular endothelial aging (47,48).

Studies have confirmed that ROS can promote endothelial cell morphological damage and are also thought to induce apoptosis (49). Necroptosis (also known as programmed necrosis) is a new way of death discovered in recent years, which is found in the pathological process of I/R injury in the heart, kidney, brain and retina. The main mechanism is that TNF-α interacts with tumor necrosis factor receptor 1 on the cell surface, which is mediated by the RIP1/RIP3/MLKL necrosis complex. Recent studies have showed that a variety of tumor cells (human lung adenocarcinoma cell line A549, human neuroblastoma cell SH-SY5Y) co-cultured with endothelial cells can induce endothelial cell necroptosis (50, 51).

**3 Repair and mechanism of damaged vascular endothelium**

Endothelial cells possess the abilities to self-proliferate and repair. Vascular endothelial injury not only affects the function of the vascular barrier, the regulatory function and secretory function of the vasodilator response, but also weakens the repair ability. Endothelial cells can slow down or even stop their own natural ageing process by reducing or preventing the damage process of endothelial cells could prevent the aging of endothelial cells，facilitating to repair damaged endothelial cells.

**3.1 Endothelial progenitor cells and endothelial repair**

Endothelial progenitor cells (endothelial progenitor cells, EPCs) are stem cells that are homed to angiogenesis tissue and could differentiate and proliferate into mature endothelial cells, and exert an important role in endothelial repair and angiogenesis. Several studies have demonstrated that the pathogenesis of various cardiovascular diseases (such as atherosclerosis, pulmonary hypertension) is associated with EPCs aging (52,53). After EPC aging, its migration, adhesion function and blood vessel formation ability are all reduced, resulting in weakened endothelial repair ability.

There are now three basic ways to treat endothelial damage with EPC: ①Transplant EPCs to endothelial injury sites to promote endothelial tissue regeneration and repair. The literature confirmed that the injection of EPC into mice could significantly improve the damage of hepatic sinusoidal endothelial cells and hepatocytes, while reducing the secretion of IL-6 and TNF-α, inhibiting platelet activation, and improving liver function (54). ②The introduction of certain genes, such as calcitonin gene-related peptide, into EPCs enhance the protective effect of EPC on endothelial cells. In animal experiments, EPCs transfected with CGRP in rats with pulmonary arterial hypertension can significantly improve pulmonary hypertension and reverse pulmonary vascular remodeling (55). In *in vitro* experiments, transfection of damaged EPCs into β2 adrenergic receptors could significantly improve the repair ability of EPCs on vascular endothelium could significantly improve the repair ability of EPC to vascular endothelium (56).③Some drugs such as low-dose aspirin, resveratrol, rosiglitazone, pyrrolizone, and evodiamine can delay EPC aging, such as resveratrol-based derivative BTM-0512 to inhibit EPC aging in diabetic rats, Its mechanism involves SIRTl-DDAH2/ADMA pathway (57); CGRP mediates Evodiamine inhibits AngⅡ-induced EPC aging, and its mechanism is related to the up-regulation of Klotho gene (58); visceral lipin delays ox-LDL-induced EPC aging and up-regulation SIRT1 is involved, and its mechanism involves the PI3K/Akt/ERK pathway (59).

**3.2 Drugs and Endothelial Protection**

Clinical studies and animal experiments have shown that many drugs, including chemical drugs and traditional Chinese medicines, have protective effects on endothelial cells, but the mechanism is not yet fully understood. Clinical studies and *in vivo* experiments have indicated that many drugs, including chemical drugs and traditional Chinese medicines, have protective effects on endothelial cells, but the mechanism is not fully understood.

The cause of this is because different studies have discussed the various entrance points of the mechanism of pharmacological protection of vascular endothelium, in addition to the complexity of the pathological mechanism of vascular endothelial injury. The drugs that protect the vascular endothelium include: ① reducing the generation of factors that induce endothelial cell damage: such as lipid-lowering drugs, hypoglycemic drugs, anti-myocardial ischemia drugs, etc. can reduce the production of blood sugar, ox-LDL, ROS, and inflammatory factors; folic acid can inhibit Hcy production, as an adjunct therapy for hyperhomocysteine-type hypertension, helps protect vascular endothelium and reduce the incidence of stroke (60); L-arginine can competitively prevent ADMA from inhibiting eNOS; vascular tension Invertase inhibitors can reduce the production of Ang II. ②The formation of ROS can be decreased by oxidative stress inhibitors such probucol, vitamin E, tanshinone IIA, etc.(61-63). ③Inhibition of inflammatory response: such as aspirin, fibrate lipid-lowering drugs, resveratrol methyl derivatives could inhibit the production of inflammatory factors (64-68). ④Delay aging of endothelial cells and EPC: such as rosiglitazone, evodiamine, simvastatin can inhibit endothelial aging (69); EPC-based transcription is regulated by epigenetic regulation, including non-coding RNA (microRNA and IncRNA), DNA methylation, histone modification (histone methylation, acetylation and deacetylation), some compounds (peptide compound inhibitor 5-azacytidine, etc.) could also pass Influencing epigenetics increases the proliferation and migration of EPC and enhances the ability to repair blood vessels (70).

**Conclusion**

Vascular endothelial injury is the initiating link of various cardiovascular and cerebrovascular diseases. In addition to the changes in its own morphology and function, endothelial injury also causes endothelial cells to secrete endogenous active substances and affect vascular smooth muscle, which affects vasodilation. On the other hand, investigations have demonstrated that a number of variables, including hypoxia and others, can cause endothelial interstitial change and promote vascular remodeling [45]. The exact mechanism of endothelial cell injury is not fully understood. Inflammation and oxidative stress are known as important pathophysiological mechanisms of endothelial injury. It is known that there is an interaction between inflammation and oxidative stress, but the network relationship of their interaction and its key molecules are yet to be elucidated. The aging and regulation mechanisms of endothelial cells and EPC also need to be further explored. With the deepening of research on endothelial injury and repair, new targets for protecting vascular endothelium will be discovered, which will provide new ideas for pharmacologists to find drugs to protect damaged endothelium. It has been proved that a variety of traditional Chinese medicines have protective effects on vascular endothelium, and the separation and purification of active ingredients and monomers in traditional Chinese medicine will be an important way to develop vascular endothelial drugs for protection.

**Authors’ Contributions**

YPH analyzed, interpreted, and presented results for group discussions. YPH and CS provided rationale, background, framework, and feedback. All authors have approved the manuscript. All authors have agreed with both to be personally accountable for the author’s contributions and to ensure that questions related to the accuracy or integrity of any part of the work.

**Funding**

This work was supported by Hunan Natural Science Foundation (grant no. 2020JJ7090.

**Conflict of Interest**

There were no conflicts of interest to be declared.

Disclaimer

None.

**Data Availability**

The data used to support the findings are available from the corresponding author upon request.

**References**

1. Bach LA. Endothelial cells and the IGF system. J Mol Endocrinol, 2015; 54(1): R1-R13.

2. Gimbrone MJ, Garcia-Cardena G. Endothelial cell dysfunction and the pathobiology of atherosclerosis. Circ Res, 2016; 118(4): 620-636.

3. Mazini L, Rochette L, Admou B, Amal S, Malka G. [Hopes and Limits of Adipose-Derived Stem Cells (ADSCs) and Mesenchymal Stem Cells (MSCs) in Wound Healing.](https://pubmed.ncbi.nlm.nih.gov/32075181/) Int J Mol Sci. 2020;21(4):1306.

4. Meng LB, Chen K, Zhang YM, Gong T.[Common Injuries and Repair Mechanisms in the Endothelial Lining.](https://pubmed.ncbi.nlm.nih.gov/30246720/) Chin Med J (Engl). 2018;131(19):2338-2345.

5. Miyamoto N, Pham LD, Seo JH, Kim KW, Lo EH, Arai K. [Crosstalk between cerebral endothelium and oligodendrocyte.](https://pubmed.ncbi.nlm.nih.gov/24132511/) Cell Mol Life Sci. 2014;71(6):1055-66.

6. [Aquiles Jara](https://pubmed.ncbi.nlm.nih.gov/?term=Jara+A&cauthor_id=19301781), [Sergio Mezzano](https://pubmed.ncbi.nlm.nih.gov/?term=Mezzano+S&cauthor_id=19301781). Vascular damage in chronic kidney disease. Rev Med Chil. 2008;136(11):1476-84.

7. Moon Y.[Predictive and Preventive Mucosal Communications in Particulate Matter Exposure-Linked Renal Distress.](https://pubmed.ncbi.nlm.nih.gov/33670188/) J Pers Med. 2021;11(2):118.

8. Stepanova TV, Ivanov AN, Tereshkina NE, Popyhova EB, Lagutina DD. [Markers of endothelial dysfunction: pathogenetic role and diagnostic significance](https://pubmed.ncbi.nlm.nih.gov/30912882/).Klin Lab Diagn. 2019;64(1):34-41.

9. Mortensen LA, Bistrup C, Stubbe J, Carlström M, Checa A, Wheelock CE, Palarasah Y, Bladbjerg EM, Thiesson HC, Jensen BL. [Effect of spironolactone for 1 yr on endothelial function and vascular inflammation biomarkers in renal transplant recipients.](https://pubmed.ncbi.nlm.nih.gov/31166706/) Am J Physiol Renal Physiol. 2019;317(3):F529-F539.

10. Arslan M, Yilmaz G, Mentese A, Yilmaz H, Karahan SC, Koksal I. [Importance of endothelial dysfunction biomarkers in patients with Crimean-Congo hemorrhagic fever.](https://pubmed.ncbi.nlm.nih.gov/28628220/) J Med Virol. 2017;89(12):2084-2091.

11. [Rinrada Kietadisorn](https://pubmed.ncbi.nlm.nih.gov/?term=Kietadisorn+R&cauthor_id=22167522), [Rio P Juni](https://pubmed.ncbi.nlm.nih.gov/?term=Juni+RP&cauthor_id=22167522), [An L Moens](https://pubmed.ncbi.nlm.nih.gov/?term=Moens+AL&cauthor_id=22167522). Tackling endothelial dysfunction by modulating NOS uncoupling: new insights into its pathogenesis and therapeutic possibilities. Am J Physiol Endocrinol Metab. 2012;302(5):E481-95.

12. Li P, Yin YL, Guo T, Sun XY, Ma H, Zhu ML, Zhao FR, Xu P, Chen Y, Wan GR, Jiang F, Peng QS, Liu C, Liu LY, Wang SX. [Inhibition of Aberrant MicroRNA-133a Expression in Endothelial Cells by Statin Prevents Endothelial Dysfunction by Targeting GTP Cyclohydrolase 1](https://pubmed.ncbi.nlm.nih.gov/27765794/) *[in Vivo](https://pubmed.ncbi.nlm.nih.gov/27765794/)*[.](https://pubmed.ncbi.nlm.nih.gov/27765794/)

Circulation. 2016;134(22):1752-1765.

13. Caliceti C, Rizzo P, Ferrari R, Fortini F, Aquila G, Leoncini E, Zambonin L, Rizzo B, Calabria D, Simoni P, Mirasoli M, Guardigli M, Hrelia S, Roda A, Cicero AFG. [Novel role of the nutraceutical bioactive compound berberine in lectin-like OxLDL receptor 1-mediated endothelial dysfunction in comparison to lovastatin.](https://pubmed.ncbi.nlm.nih.gov/28511903/) Nutr Metab Cardiovasc Dis. 2017;27(6):552-563.

14. Yang C, Zhao Y, Ren D, Yang X. [Protective Effect of Saponins-Enriched Fraction of Gynostemma pentaphyllum against High Choline-Induced Vascular Endothelial Dysfunction and Hepatic Damage in Mice.](https://pubmed.ncbi.nlm.nih.gov/32115504/) Biol Pharm Bull. 2020;43(3):463-473.

15. Nègre-Salvayre A, Garoby-Salom S, Swiader A, Rouahi M, Pucelle M, Salvayre R.[Proatherogenic effects of 4-hydroxynonenal.](https://pubmed.ncbi.nlm.nih.gov/28040472/) Free Radic Biol Med. 2017;111:127-139.

16.[Francesca Schinzari](https://pubmed.ncbi.nlm.nih.gov/?term=Schinzari+F&cauthor_id=28141700), [Manfredi Tesauro](https://pubmed.ncbi.nlm.nih.gov/?term=Tesauro+M&cauthor_id=28141700), [Carmine Cardillo](https://pubmed.ncbi.nlm.nih.gov/?term=Cardillo+C&cauthor_id=28141700). Endothelial and Perivascular Adipose Tissue Abnormalities in Obesity-Related Vascular Dysfunction: Novel Targets for Treatment. J Cardiovasc Pharmacol. 2017;69(6):360-368.

17. Zhong S, Li L, Shen X, Li Q, Xu W, Wang X, Tao Y, Yin H.[An update on lipid oxidation and inflammation in cardiovascular diseases.](https://pubmed.ncbi.nlm.nih.gov/30946962/) Free Radic Biol Med. 2019;144:266-278.

18. Ross R. Atherosclerosis--an inflammatory disease. N Engl J Med, 1999, 340(2): 115-126.

19. Jiang SJ, Tsai PI, Peng SY, Chang CC, Chung Y, Tsao HH, Huang HT, Chen SY, Hsu HJ. [A potential peptide derived from cytokine receptors can bind proinflammatory cytokines as a therapeutic strategy for anti-inflammation.](https://pubmed.ncbi.nlm.nih.gov/30783144/) Sci Rep. 2019;9(1):2317.

20. [Fan W, Cheng K, Qin X, Narsinh KH, Wang S, Hu S, Wang Y, Chen Y, Wu JC, Xiong L, Cao F. mTORC1 and mTORC2 play different roles in the functional survival of transplanted adipose-derived stromal cells in hind limb ischemic mice via regulating inflammation in vivo.](https://pubmed.ncbi.nlm.nih.gov/23081858/)Stem Cells. 2013;31(1):203-14.

21. Bai B, Yang Y, Wang Q, Li M, Tian C, Liu Y, Aung LHH, Li PF, Yu T, Chu XM. [NLRP3 inflammasome in endothelial dysfunction.](https://pubmed.ncbi.nlm.nih.gov/32948742/) Cell Death Dis. 2020;11(9):776.

22. Domingueti CP, Dusse LM, Carvalho Md, de Sousa LP, Gomes KB, Fernandes AP. [Diabetes mellitus: The linkage between oxidative stress, inflammation, hypercoagulability and vascular complications.](https://pubmed.ncbi.nlm.nih.gov/26781070/) J Diabetes Complications. 2016;30(4):738-45.

23. [Jose Gabriel Solis](https://pubmed.ncbi.nlm.nih.gov/?term=Solis+JG&cauthor_id=33972118), [Ervin Saul Enciso López](https://pubmed.ncbi.nlm.nih.gov/?term=Enciso+L%C3%B3pez+ES&cauthor_id=33972118), [Aleida Bautista Santos](https://pubmed.ncbi.nlm.nih.gov/?term=Bautista+Santos+A&cauthor_id=33972118), [Juan Carlos Anda Garay](https://pubmed.ncbi.nlm.nih.gov/?term=Anda+Garay+JC&cauthor_id=33972118), [Jose Luis Calixto Rodríguez](https://pubmed.ncbi.nlm.nih.gov/?term=Calixto+Rodr%C3%ADguez+JL&cauthor_id=33972118), [Rosalba Moreno Alcántar](https://pubmed.ncbi.nlm.nih.gov/?term=Moreno+Alc%C3%A1ntar+R&cauthor_id=33972118), [Luis Montiel López](https://pubmed.ncbi.nlm.nih.gov/?term=Montiel+L%C3%B3pez+L&cauthor_id=33972118). Effect of Antiviral Agents on Atherosclerosis in Patients with Chronic Hepatitis C. Arch Med Res. 2021;52(7):764-771.

24. Pleskovič A, Letonja MŠ, Vujkovac AC, Nikolajević Starčević J, Gazdikova K, Caprnda M, Gaspar L, Kruzliak P, Petrovič D. [C-reactive protein as a marker of progression of carotid atherosclerosis in subjects with type 2 diabetes mellitus.](https://pubmed.ncbi.nlm.nih.gov/28218562/)Vasa. 2017;46(3):187-192.

25. Forouzanfar F, Asgharzade S. [MicroRNAs in Noise-Induced Hearing Loss and their Regulation by Oxidative Stress and Inflammation.](https://pubmed.ncbi.nlm.nih.gov/32538724/)Curr Drug Targets. 2020;21(12):1216-1224.

26. Wu YL, Li HF, Chen HH, Lin H. [MicroRNAs as Biomarkers and Therapeutic Targets in Inflammation- and Ischemia-Reperfusion-Related Acute Renal Injury.](https://pubmed.ncbi.nlm.nih.gov/32937906/) Int J Mol Sci. 2020;21(18):6738.

27. Luan X, Zhou X, Fallah P, Pandya M, Lyu H, Foyle D, Burch D, Diekwisch TGH. [MicroRNAs: Harbingers and shapers of periodontal inflammation.](https://pubmed.ncbi.nlm.nih.gov/34120836/) Semin Cell Dev Biol. 2022;124:85-98.

28. [Olivia de Montgolfier](https://pubmed.ncbi.nlm.nih.gov/?term=de+Montgolfier+O&cauthor_id=31093829), [Philippe Pouliot](https://pubmed.ncbi.nlm.nih.gov/?term=Pouliot+P&cauthor_id=31093829), [Marc-Antoine Gillis](https://pubmed.ncbi.nlm.nih.gov/?term=Gillis+MA&cauthor_id=31093829), [Guylaine Ferland](https://pubmed.ncbi.nlm.nih.gov/?term=Ferland+G&cauthor_id=31093829), [Frédéric Lesage](https://pubmed.ncbi.nlm.nih.gov/?term=Lesage+F&cauthor_id=31093829), [Nathalie Thorin-Trescases](https://pubmed.ncbi.nlm.nih.gov/?term=Thorin-Trescases+N&cauthor_id=31093829), [Éric Thorin](https://pubmed.ncbi.nlm.nih.gov/?term=Thorin+%C3%89&cauthor_id=31093829). Systolic hypertension-induced neurovascular unit disruption magnifies vascular cognitive impairment in middle-age atherosclerotic LDLr-/-:hApoB+/+mice. Geroscience. 2019;41(5):511-532.

29. Zhu N, Hou J. [Molecular mechanism of the anti-inflammatory effects of Sophorae Flavescentis Aiton identified by network pharmacology.](https://pubmed.ncbi.nlm.nih.gov/33441867/) Sci Rep. 2021;11(1):1005.

30. Wahid A, Chen W, Wang X, Tang X. [High-mobility group box 1 serves as an inflammation driver of cardiovascular disease.](https://pubmed.ncbi.nlm.nih.gov/33865014/) Biomed Pharmacother. 2021;139:111555.

31. Jeong J, Lee J, Lim J, Cho S, An S, Lee M, Yoon N, Seo M, Lim S, Park S. [Soluble RAGE attenuates AngII-induced endothelial hyperpermeability by disrupting HMGB1-mediated crosstalk between AT1R and RAGE.](https://pubmed.ncbi.nlm.nih.gov/31562296/) Exp Mol Med. 2019;51(9):1-15.

32. Hebbel RP, Wei P, Milbauer L, Corban MT, Solovey A, Kiley J, Pattee J, Lerman LO, Pan W, Lerman A [Abnormal Endothelial Gene Expression Associated With Early Coronary Atherosclerosis.](https://pubmed.ncbi.nlm.nih.gov/32673514/).J Am Heart Assoc. 2020;9(14):e016134.

33. Florim GMS, Caldas HC, Gonçalves NN, Bueno GOBE, Baptista MASF, Fernandes-Charpiot IMM, Abbud-Filho M. [Activation of HMGB1-TLR4 Pathway and Inflammasome Contribute to Enhanced Inflammatory Response in Extended Criteria and Kidneys With KDPI ≥85.](https://pubmed.ncbi.nlm.nih.gov/31764760/) Transplantation. 2020 ;104(4):724-730.

34. Palomo J, Dietrich D, Martin P, Palmer G, Gabay C. [The interleukin (IL)-1 cytokine family--Balance between agonists and antagonists in inflammatory diseases.](https://pubmed.ncbi.nlm.nih.gov/26185894/) Cytokine. 2015;76(1):25-37.

35. [Je-Wook Yu](https://pubmed.ncbi.nlm.nih.gov/?term=Yu+JW&cauthor_id=27600432), [Myung-Shik Lee](https://pubmed.ncbi.nlm.nih.gov/?term=Lee+MS&cauthor_id=27600432). Mitochondria and the NLRP3 inflammasome: physiological and pathological relevance. Arch Pharm Res. 2016 Nov;39(11):1503-1518.

36. Dinarello CA. [Interleukin-1 in the pathogenesis and treatment of inflammatory diseases.](https://pubmed.ncbi.nlm.nih.gov/21304099/) Blood. 2011;117(14):3720-32.

37. Fahey E, Doyle SL. [IL-1 Family Cytokine Regulation of Vascular Permeability and Angiogenesis.](https://pubmed.ncbi.nlm.nih.gov/31293586/)Front Immunol. 2019; 10:1426.

38. Ungvári Z, Gupte SA, Recchia FA, Bátkai S, Pacher P. [Role of oxidative-nitrosative stress and downstream pathways in various forms of cardiomyopathy and heart failure.](https://pubmed.ncbi.nlm.nih.gov/16026319/) Curr Vasc Pharmacol. 2005;3(3):221-9.

39. Husain K, Hernandez W, Ansari RA, Ferder L. [Inflammation, oxidative stress and renin angiotensin system in atherosclerosis.](https://pubmed.ncbi.nlm.nih.gov/26322175/) World J Biol Chem. 2015;6(3):209-17.

40. Shaw A, Doherty MK, Mutch NJ, et al. Endothelial cell oxidative stress in diabetes: a key driver of cardiovascular complications?. Biochem Soc Trans, 2014; 42(4): 928-933.

41. Tan B, Jiang DJ, Huang H, et al. Taurine protects against low-density lipoprotein-induced endothelial dysfunction by the DDAH/ADMA pathway. Vascul Pharmacol, 200; 46(5): 338-345.

42. Jiang DJ, Jia SJ, Yan J, et al. Involvement of DDAH/ADMA/NOS pathway in nicotine-induced endothelial dysfunction. Biochem Biophys Res Commun, 2006;349(2): 683-693.

43. Yuan Q, Hu CP, Gong ZC, et al. Accelerated onset of senescence of endothelial progenitor cells in patients with type 2 diabetes mellitus: role of dimethylarginine dimethylaminohydrolase 2 and asymmetric dimethylarginine. Biochem Biophys Res Commun, 2015; 458(4): 869-876.

44. O'Flaherty C.[Orchestrating the antioxidant defenses in the epididymis.](https://pubmed.ncbi.nlm.nih.gov/31044545/) Andrology. 2019;7(5):662-668.

45. Awad MA, Aldosari SR, Abid MR. [Genetic Alterations in Oxidant and Anti-Oxidant Enzymes in the Vascular System.](https://pubmed.ncbi.nlm.nih.gov/30140678/) Front Cardiovasc Med. 2018;5:107.

46. Bai YP, Hu CP, Yuan Q, et al. Role of VPO1, a newly identified heme-containing peroxidase, in ox-LDL induced endothelial cell apoptosis. Free Radic Biol Med, 2011;51(8): 1 492-500.

47. Ma QL, Zhang GG, Peng J. Vascular peroxidase 1: a novel enzyme in promoting oxidative stress in cardiovascular system. Trends Cardiovasc Med, 2013; 23(5): 179-183.

48.Ishii M, Kaikita K, Sakamoto K, Seki T, Kawakami K, Nakai M, Sumita Y, Nishimura K, Miyamoto Y, Noguchi T, Yasuda S, Tsutsui H, Komuro I, Saito Y, Ogawa H, Tsujita K; JROAD Investigators. [Characteristics and in-hospital mortality of patients with myocardial infarction in the absence of obstructive coronary artery disease in super-aging society.](https://pubmed.ncbi.nlm.nih.gov/31740139/) Int J Cardiol. 2020;301:108-113.

49. Tian XL, Li Y. Endothelial cell senescence and age-related vascular diseases. J Genet Genomics, 2014; 41(9): 485-495.

50. Liu SY, Yuan Q, Li XH, et al. Role of vascular peroxidase 1 in senescence of endothelial cells in diabetes rats. Int J Cardiol, 2015; 197: 182-191.

51.Yang W, Liu Z, Xu Q, Peng H, Chen L, Huang X, Yang T, Yu Z, Cheng G, Zhang G, Shi R. [Involvement of vascular peroxidase 1 in angiotensin II-induced hypertrophy of H9c2 cells.](https://pubmed.ncbi.nlm.nih.gov/27651140/) J Am Soc Hypertens. 2017;11(8):519-529.e1.

52.Shokrzadeh M, Bagheri A, Ghassemi-Barghi N, Rahmanian N, Eskandani M. [Doxorubicin and doxorubicin-loaded nanoliposome induce senescence by enhancing oxidative stress, hepatotoxicity, and in vivo genotoxicity in male Wistar rats.](https://pubmed.ncbi.nlm.nih.gov/34219194/) Naunyn Schmiedebergs Arch Pharmacol. 2021;394(8):1803-1813.

53.Xiao L, Gu Y, Ren G, Chen L, Liu L, Wang X, Gao L. [miRNA-146a Mimic Inhibits NOX4/P38 Signalling to Ameliorate Mouse Myocardial Ischaemia Reperfusion (I/R) Injury.](https://pubmed.ncbi.nlm.nih.gov/34367463/) Oxid Med Cell Longev. 2021;2021:6366254.

54.Matacchione G, Gurău F, Silvestrini A, Tiboni M, Mancini L, Valli D, Rippo MR, Recchioni R, Marcheselli F, Carnevali O, Procopio AD, Casettari L, Olivieri F. [Anti-SASP and anti-inflammatory activity of resveratrol, curcumin and β-caryophyllene association on human endothelial and monocytic cells.](https://pubmed.ncbi.nlm.nih.gov/33704623/) Biogerontology. 2021;22(3):297-313.

55. Zhang C, Wang H, Yang B. [miR-146a regulates inflammation and development in patients with abdominal aortic aneurysms by targeting CARD10.](https://pubmed.ncbi.nlm.nih.gov/32138469/) Int Angiol. 2020;39(4):314-322.

56. Strilic B, Yang L, Albarran-Juarez J, et al. Tumour-cell-induced endothelial cell necroptosis via death receptor 6 promotes metastasis. Nature, 2016; 536(7615): 215-218.

57. Lubrano V, Balzan S. LOX-1 and ROS, inseparable factors in the process of endothelial damage. Free Radic Res, 2014, 48(8): 841-848.

58. Chen D, Yu J, Zhang L. Necroptosis: an alternative cell death program defending against cancer. Biochim Biophys Acta, 2016;1865(2): 228-236.

59. [Zhi Zhou](https://pubmed.ncbi.nlm.nih.gov/?term=Zhou+Z&cauthor_id=20832068), [Chang-Ping Hu](https://pubmed.ncbi.nlm.nih.gov/?term=Hu+CP&cauthor_id=20832068), [Chen-Jing Wang](https://pubmed.ncbi.nlm.nih.gov/?term=Wang+CJ&cauthor_id=20832068), [Ting-Ting Li](https://pubmed.ncbi.nlm.nih.gov/?term=Li+TT&cauthor_id=20832068), [Jun Peng](https://pubmed.ncbi.nlm.nih.gov/?term=Peng+J&cauthor_id=20832068), [Yuan-Jian Li](https://pubmed.ncbi.nlm.nih.gov/?term=Li+YJ&cauthor_id=20832068). Calcitonin gene-related peptide inhibits angiotensin II-induced endothelial progenitor cells senescence through up-regulation of klotho expression Atherosclerosis, 2010; 213(1): 92-101.

60. Zhou Z, Peng J, Wang CJ, et al. Accelerated senescence of endothelial progenitor cells in hypertension is related to the reduction of calcitonin gene-related peptide. J Hypertens, 2010; 28(5): 931-939.

61. Qiao J, Qi K, Chu P, et al. Infusion of endothelial progenitor cells ameliorates liver injury in mice after haematopoietic stem cell transplantation. Liver Int, 2015;35(12): 2 611-620.

62. Zhao Q, Liu Z, Wang Z, et al. Effect of prepro-calcitonin gene-related peptide-expressing endothelial progenitor cells on pulmonary hypertension. Ann Thorac Surg, 2007;84(2): 544-552.

63. Ke X, Shu XR, Wu F, et al. Overexpression of the beta 2AR gene improves function and re-endothelialization capacity of EPCs after arterial injury in nude mice. Stem Cell Res Ther, 2016; 7(1): 73.

64. Yuan Q, Hu CP, Gong ZC, et al. Accelerated onset of senescence of endothelial progenitor cells in patients with type 2 diabetes mellitus: role of dimethylarginine dimethylaminohydrolase 2 and asymmetric dimethylarginine. Biochem Biophys Res Commun, 2015;458(4): 869-876.

65. Zhou Z, Hu CP, Wang CJ, et al. Calcitonin gene-related peptide inhibits angiotensin II-induced endothelial progenitor cells senescence through up-regulation of klotho expression. Atherosclerosis, 2010;213(1): 92-101.

66.Ming GF, Tang YJ, Hu K, et al. Visfatin attenuates the ox-LDL-induced senescence of endothelial progenitor cells by upregulating SIRT1 expression through the PI3K/Akt/ERK pathway. Int J Mol Med, 2016;38(2): 643-649.

67.Xu S, Ilyas I, Little PJ, Li H, Kamato D, Zheng X, Luo S, Li Z, Liu P, Han J, Harding IC, Ebong EE, Cameron SJ, Stewart AG, Weng J. [Endothelial Dysfunction in Atherosclerotic Cardiovascular Diseases and Beyond: From Mechanism to Pharmacotherapies.](https://pubmed.ncbi.nlm.nih.gov/34088867/) Pharmacol Rev. 2021;73(3):924-967.

68. Yang F, Tan HM, Wang H. Hyperhomocysteinemia and atherosclerosis. Sheng Li Xue Bao, 2005;57(2): 103-114.

69. Haklar G, Sirikci O, Ozer NK, et al. Measurement of reactive oxygen species by chemiluminescence in diet-induced atherosclerosis: protective roles of vitamin E and probucol on different radical species. Int J Clin Lab Res, 1998;28(2): 122-126.

70. Tang C, Xue HL, Bai CL, et al. Regulation of adhesion molecules expression in TNF-α-stimulated brain microvascular endothelial cells by tanshinone IIA: involvement of NF-kappa B and ROS generation. Phytother Res, 2011;25(3): 376-380.

71. Jia LQ, Yang GL, Ren L, et al. Tanshinone IIA reduces apoptosis induced by hydrogen peroxide in the human endothelium-derived EA.hy926 cells. J Ethnopharmacol, 2012;143(1): 100-108.

72. Tsai KL, Huang PH, Kao CL, et al. Aspirin attenuates vinorelbine-induced endothelial inflammation via modulating SIRT1/AMPK axis. Biochem Pharmacol, 2014; 88(2): 189-200.

73. Price ET, Welder GJ, Zineh I. Modulatory effect of fenofibrate on endothelial production of neutrophil chemokines IL-8 and ENA-78. Cardiovasc Drugs Ther, 2012; 26(2): 95-99.

74. Wang W, Bai L, Qiao H, et al. The protective effect of fenofibrate against TNF-α-induced CD40 expression through SIRT1-mediated deacetylation of NF-kappa B in endothelial cells. Inflammation, 2014; 37(1): 177-185.

75. Jeong SO, Son Y, Lee JH, et al. Resveratrol analog piceatannol restores the palmitic acid-induced impairment of insulin signaling and production of endothelial nitric oxide via activation of anti-inflammatory and antioxidative heme oxygenase-1 in human endothelial cells. Mol Med Rep, 2015;12(1): 937-944.

76. Pan W, Yu H, Huang S, et al. Resveratrol protects against TNF-α-induced injury in human umbilical endothelial cells through promoting sirtuin-1-induced repression of NF-KB and p38 MAPK. PLoS One, 2016; 11(1): e147 034.

77.Han X, Jiang G, Shi Q.[Effects of antihyperglycemics on endothelial progenitor cells.](https://pubmed.ncbi.nlm.nih.gov/33210492/) Zhejiang Da Xue Xue Bao Yi Xue Ban. 2020 25;49(5):629-636.

78. [Sylvain Fraineau](https://pubmed.ncbi.nlm.nih.gov/?term=Fraineau+S&cauthor_id=25546332), [Carmen G Palii](https://pubmed.ncbi.nlm.nih.gov/?term=Palii+CG&cauthor_id=25546332), [David S Allan](https://pubmed.ncbi.nlm.nih.gov/?term=Allan+DS&cauthor_id=25546332), [Marjorie Brand](https://pubmed.ncbi.nlm.nih.gov/?term=Brand+M&cauthor_id=25546332). Epigenetic regulation of eothelial-cell-mediated vascular repair. FEBS J, 2015; 282(9): 1 605-629.
